# Supplementary material for: Orientation processing by synaptic integration across first-order tactile neurons
Source: PLoS Comput Biol. 2020 Dec 2;16(12):e1008303. doi: 10.1371/journal.pcbi.1008303 (PMC7710081; doi:10.1371/journal.pcbi.1008303)
Supplement: Attachment — Submitted filename: Hay 2020 - response to reviewers.pdf [file pcbi.1008303.s001.pdf]

Reviewer #1: The paper describes the development and optimization of a model of first order spiking sensory neuron responding to tactile stimuli of varying orientations. Optimization is carried out by varying mechanoreceptor location and their receptive fields to fit human neuronal recordings. Ultimately, the fitted model is claimed to be predictive for unforeseen stimuli.

Having the model for spiking patterns devised, the authors created a classifier that relies on population of modeled neurons to determine the stimulus orientation. In this context, the difference between slowly integrating NMDA based classifier and a fast AMPA based one is demonstrated with AMPA synapses outperforming NMDA with high resolution orientation detection.

We thank the reviewer for their very useful comments.

Major comments:

1. The model was trained on non-trivial positive/negative angles and then tested only on a zero angle. It is recommended that the authors will test the model against several angles and compare and analyze the dependency of model performance on stimulus angle. Furthermore, from the methods if sounds like the test set (angle zero) was used in the final model picking. This should be clarified to ensure appropriate separation between fitting and cross validation. Particularly alarming is the statement at the bottom of page 18 saying that the model was selected based, among other things, on its test performance.

According to the reviewer's comment, we have clarified the model optimization/cross-validation process, and added a proper testing method. The 0˚ edge was used for cross-validation, and we specify it more clearly now. We have introduced a new test data (edges oriented 30˚) for test prediction accuracy, and revised accordingly (Figure 1, results p. 3-4, methods p.15-16). The prediction accuracy of the models on test data was similar to their cross-validation accuracy. In addition, we calculated the experimental response reliability for the test orientation (R^2 over the different trials of each recorded neuron's response), and show that the model prediction accuracy is close to the experimental response reliability (Figure 1, results p. 4).

2. Another point, which claimed to be a major advance (in intro/discussion), is relying on the actual spike trains for modeling. It should be clarified what part of the modeling process will fail if a model based on rates or on first neuron to spike or other less detailed description of neuronal activity is considered. In particular, given an integrator that the authors use at the input to the classifier essentially performs a translation from spikes to rate..

According to the reviewer's suggestion, we have added to the discussion of this point another example of the benefit of spiking in the model, namely the model's ability to reproduce the sharply varying spatiotemporal stimulus response as seen by the experimental firing rate curve, which would increase the neuron's computational power (discussion, p.12). The previous simpler model that used analogue convolution was limited in the sharp transitions in responsivity. We also note that the spatiotemporally distinct events of high frequency response are also expected to translate to a sharply differing synaptic summation despite the smoothing effect of the synapses.

3. The authors claim that the AMPA based model works like a coincidence detector, whereas the NMDA based model "may mediate the discrimination of objects based on their macro-geometric features". Beyond Fig3A-C, the comparative results are based on accuracy in the presence of additive noise, which the authors acknowledge to be a minor issue for tactile perception. To make the case of AMPA vs. NMDA strong, and given the central role noise plays in the comparison, it is recommended to:
- explain what noise level was used for training. If training was done with zero noise, please redo it with the noise level at which Fig 3 is presented (i.e. 10%).
- describe the noise protocol in more details (in Methods), including noise spectrum and the way the non-negativity is enforced.
- repeat FIG4 for the NMDA receptive fields, and further repeat it for fine orientations (+/-1 or +/-3deg) to emphasize the synaptic mechanism of coincidence detection.
- analyze further the deterioration of AMPA results for long integration time (in Fig. 3A). - quantify the Signal-to-Noise ratio at the classifier's input as a function of the integration time

- For each noise level examined, classifiers were trained with training data that had the same level of noise as the data on which they were tested. We clarified this point and described the noise level in further detail in the Methods (p.17).
- According to the reviewer's suggestion, we have repeated Figure 4 for the NMDA classifier receptive fields, and generated a new figure (Figure 5). We used this new figure to illustrate the difference in classification using coincidence detection vs. summation. The difference was evident in the narrow vs. broad RF areas of responsivity of the classifier units that used AMPA vs. NMDA synapses (results, p.7-8, Figure 5).
- For fine orientations, the receptive field plots were not as informative because the stimulus did not pass over much of the skin patch. We therefore focused on the above new figure to illustrate the different integration mechanisms.
- The signal-to-noise ratio at the classifier's input was constant for a given noise level regardless of the time window, therefore the challenge for AMPA integration in longer time windows was more due to the difficulty in finding the inputs that robustly coincided for the given edge orientation and did not coincide by chance for the other orientation. We have added a comment to this effect in the discussion (p. 10).

4. The model is an open-loop model. In typical tactile sensing, humans control their

scanning kinematics, probably as a function of the tactile input (a closed-loop control). The authors should refer to the difference between these two modes of perception and discuss how their results can contribute to the understanding of closed-loop tactile perception.

The reviewer is correct to point out that humans move their hands relative to objects and this something that we and others have studied in detail. We are now more explicit on how our results relate to closed-loop control (discussion, p.11).

Minor points:

1. In the discussion it is suggested that AMPA receptors allow fast discrimination while NMDA receptors are responsible for more fine tuning-but the results suggest that that AMPA rectors are superior to NMDA at short time scales for small angles only (fig.2E.fig.3C,F).

Agreed. According to the reviewer's suggestion, we revised to say more specifically that the AMPA integration allows for fast and robust discrimination of fine angles, and that NMDA integration supported discrimination of non-fine angles over short time windows and maintained robustness of discrimination over long time windows (discussion, p.10; abstract, p.1).

2. The change in performance with time is related to AMPA and NMDA decay constants-how does these relate to the frequency of stimulus presentation (and possible adaptation affects).

We added a discussion of this point, specifying that indeed the fast kinetics of AMPA enable it to differentially respond to brief stimuli, and reduced their robustness in sustaining discrimination over longer time windows (discussion, p.10).

3. Comparison to ref. 6 (page 15): what figure of merit is compared?

This discussion point is primarily regarding the prediction of response firing rate in our models compared to previous simpler models. We revised the discussion to clarify:
Compared to previous simpler models that predicted the average response firing rate using convolution of the recorded receptive field responsivity [ref 6], our model neurons enabled a good prediction accuracy of single-trial response spiking rate and included physiological detail of mechanoreceptor innervation and spiking. We then proceeded with detailed benefits of the spiking model (p.12).

4. Argument for sparseness in figure 4A: Does that mean that the majority of 330 neurons are redundant? Or are they used for fine orientations (e.g. +/-1deg.).

We have added to the discussion a point about this: The reliance on a small set of key inputs can enable utilizing inputs from the other neurons for discriminating different orientations or stimulus features, as is also demonstrated in our two-orientations task by the different sets of neurons contributing to discrimination of the different orientations (p.11).

Reviewer #2: In this manuscript, Etay and Pruszynski built spiking models of first-order tactile neurons and explored if/how a decoder extracts orientation information from a population of such neurons. The first-order neurons have complex receptive fields that endow them with orientation tuning. I think the results are interesting and, from what I can tell, the modeling has been conducted carefully and thoroughly. I do, however, have some concerns and suggestions for how the improve the paper.

We thank the reviewer for their very useful comments and for appreciating our results.

Main concerns:

-The Introduction explains that both FA-1 and SA-1 neurons play a role in sensing fine spatial details. But for the rest of the paper, only FA-1 neurons are considered. This leaves me wondering how the FA-1 and SA-1 neurons might differ, and specifically whether they contribute differentially to orientation tuning (or would be decoded differently). Especially when considering the timescales of AMPA and NMDA synapses, I'm wondering if the timescale of SA-1 responses (if different from FA-1 responses) would cause decoding to be better/worse for AMPA/NMDA synapses. At the very least, there should be some discussion of these issues.

This is an interesting point, and at the reviewer's suggestion we have added a discussion of this point (discussion, p.12). Due to the similar transient high-frequency response of SA-1 neurons, which enables them to report edge-orientations, we expect AMPA integration to be similar for both neurons. It is possible that NMDA integration of SA-1 inputs will decrease the discrimination accuracy due to their sustained firing, but since the sustained rate is low the effect may not be large.

- I'm unclear whether the reset scheme used to model spike generation across different axon branches is critical. Is that the only scheme that works to reproduce the experimental spike trains, or do alternative schemes (e.g. summation) also work? If both work, are there any implications for subsequent decoding?

We have focused on the reset scheme as previous empirical studies including our own suggest that it is most likely for this class of neurons at least for these types of stimuli. This we note in the introduction and the relevant papers are cited in the Methods. Nevertheless, over the modeling course, we did examine two alternative spiking schemes to the reset scheme:

1. Models with spike mixing were not able to reproduce the experimental spike trains well, since they tended to have high firing rate throughout the response (a higher number of spikes due to a lack of the toning-down effect of spike reset).
2. Summation worked better than mixing, but not as good as the reset scheme. Models that used summation did not produce the sharp transitions in firing response rate curve seen experimentally.
We have added this information in the discussion (p. 12).

-The authors developed 15 different neurons for primary afferent neurons. A patch of skin is modeled using 330 models, each of which is randomly rotated. This means that each model is used 22 (or more) times. Notwithstanding the rotation, I worry that re-using identical models might introduce unintended structure that would not occur naturally and which might affect decoding. I suggest morphing the existing models so that they have the same RF structure in a statistical sense, but are not identical, and conducting some test simulations to rule out key differences from the random rotation approach. Alternatively, would building a 330-cell network with just one sample neuron (instead of 15, but still rotating them), yield equivalent results to the current approach. If the test simulation don't raise concerns, I do NOT see any need to repeat all the simulations.

Our results were similar even when using a smaller set of model neurons (e.g. 8) to create the population. In addition, Figure 6 (new) shows the response of several neurons in the population and includes a pair of model neurons that are rotated versions of a single model neuron. The response of such rotated variations was different in firing pattern, peak firing rate, and duration, attesting to the diversity of the model first-order neuronal population. We have added a point to that effect in the discussion (p. 13).

-The issue of a stimulation time window raised questions for me. The RF is mapped experimentally by sweeping a single edge (or grating?) across the RF, correct? If so, does the "time window" relate to the sweep speed? There is room to clarify how the stimulus is implemented in the model and what certain parameters mean in a physiological sense.

We clarified in the methods that the data and simulations involved a single edge at a time, moving over the receptive field at a speed of 30 mm/s (methods, p.14, p.17).
We also clarified that in the simulations the stimulus presentation time window did not involve a change in stimulus speed, but rather referred to how long the stimulus was presented around the center of the skin patch. Longer presentation time windows meant that edge passed over larger parts of the skin patch, whereas shorter presentation time windows meant that the edge passed over a smaller part of the skin patch around its center. We added a clarification to this effect (methods, p.17-18).

-Figure 4 explains that only a small number of sensory neurons (8 to 11, out of 330) are used to decode orientation as inferred from synaptic weights. It is mentioned that the weights of key synapses are anticorrelated between classifiers tuned to opposite

orientations. This leads me to suspect that all the sensory neurons providing input to each classifier (i.e. synapses with significant weight) all have the same orientation tuning, and the classifier simply inherits that tuning. In other words, optimizing the synaptic weight amounts to finding the correctly tuned sensory neurons, connecting to them, and not connecting to sensory neurons with other tuning. Is that correct? If so, the result seems a little trivial. More sophisticated downstream circuitry (e.g. with inhibition driven by sensory neurons with orthogonal orientation tuning) might refine the decoding, but this isn't considered. Please comment.

This is a good point. We have investigated the key neurons and generated a new figure (Figure 6), where we show that the key neurons are highly varied in their response and tuning selectivity. The model neurons also stem from different model neurons from our set of 15 neurons, varying in RF size and innervation pattern. We give example of neurons that were not tuned to the orientation in either peak firing rate or firing duration. In addition, we show that rotated variations from the same source model neuron resulted in model neurons with largely differing tuning. Thus, the edge-orientation discrimination depended on synaptic integration over model neurons with different tuning (Figure 6; results, p.8-9).

Other points:
-Articles like "the" or "a" are often missing. Examples (p 3): with AN axon spike initiation zone; we used A genetic algorithm; best fit of THE model neuron. There is room to improve the writing in this regard.

We have revised throughout the manuscript to include these articles.

-I do not see any supplementary files providing the data. More importantly for this sort of paper, I do not see any statement about the availability of the code used for modeling.

We will make the code available online upon publication. We have added a statement that the code is available online on ModelDB (p.18).

Reviewer #3: The receptive fields of first order tactile neurons have multiple hotspots. In a previous paper, the senior author has proposed that this receptive field structure contributes to the extraction of the geometric features of a stimulus, namely edge orientation. This new study first develops a simple model of the response of individual first order neurons. The main innovation in the model is that it incorporates multiple mechanoreceptors, whose spiking response is then integrated using a winner-take-all mechanism. This model can faithfully reproduce the responses of first order neurons to scanned edges. Second, the response of a population of first order neurons to scanned edges is simulated using the model. The output of the neurons is then convolved with one of two filters – one designed to mimic fast decaying AMPA-mediated currents, the other designed to mimic slow decaying NMDA-mediated currents. The output of the resulting population of simulated second order neurons is then used to classify the orientation of

the stimulus. Classification performance is found to depend on stimulus duration and noise and also depends on which filter is used to generate the second order signals.

How first order neurons integrate signals from different mechanoreceptors is an interesting and important question, which this study purports to address. However, this aspect of the study is then combined with a different one, which is to assess whether orientation can be decoded from these simulated signals. Neither model – encoding or decoding – is validated. This work rather thus constitutes a proof of principle rather than a demonstration.

We thank the reviewer for their comments.

Major comments

1. The model is fit to scanned edges and tested on scanned edges. It is difficult to evaluate the performance of the model and compare it to previous models. For example, previous models of first order neurons achieved comparable performance in predicting neural responses to a wide variety of stimuli, including edges. How do the models compare in terms of performance? This is important to establish the importance of the multi-receptor models (the main innovation in the encoding model) compared to single-receptor models.

It is important to emphasize that out prime interest was not comparing single-receptor to multi-receptor models since we know that empirically these neurons have multiple receptors. Rather, our prime interest was to understand how second order neurons operate given the complex nature of the inputs they receive. That said, the reviewer makes an excellent suggestion to better tie these ideas to past efforts. Therefore, we have added a new figure (Figure 7, p.9) that compared edge-orientation discrimination accuracy using a population of neurons that had a simple receptive field (a single mechanoreceptor, with similar responsivity profile as employed in the multiple-mechanoreceptor model). The model thus exhibited a radius of low excitability and a small center of higher excitability, and functioned qualitatively similar to previous models with simple receptive field.

We show that edge-discrimination of fine orientations is reduced when integrating AMPA or NMDA synaptic inputs from neurons with simple receptive field. For AMPA synapses, a simple receptive field further reduced the robustness of discrimination accuracy over long stimulus presentation time windows. For NMDA, using a simple receptive field had a comparable robustness to complex receptive field.

Overall, the simple receptive field reduced the ability to discriminate fine edge-orientations, regardless of the type of synapse or stimulus presentation time window. In addition, we were able to delineate some cases where the simple receptive field performed comparably (e.g. when discriminating coarser orientations).

We have also added a discussion point regarding these points and possible future directions in comparing the utility of the complex RF, e.g. using more difficult discrimination tasks (p.13-14).

2. One of the questions about coding in first order neurons is the extent to which these responses convey stimulus information in a reliable way. In this study, all the edges are scanned at a single speed. Robustness to noise is tested, with Gaussian noise. The dependence of the (simulated) neural responses to noise are poorly characterized. What happens when you change the size of the noise patch, e.g.? Only the consequence of the noise on decoding performance is discussed. The noise seems to be largely arbitrary (Why .4 x .4 mm patches? for example). More importantly, to what extent would the models be able to accommodate edges with different geometries, scanned at different speeds (on which the senior author has previously published), or indented into the skin? How to first order neurons respond to these stimuli? Can the orientation of these stimuli be decoded using a single decoder?

We clarified that the size of the noise patch was chosen as .4 x .4 mm to correspond to the smallest stimulus (dot) in the experimental data. In addition the choice was reasonable in terms of surface irregularities (methods, p.17). It could indeed be interesting to study the effects of different types of noise, but we chose to focus on varying the noise level and believe that further variations may unnecessarily detract from the focus of the current study. We have added a point discussing this as potential future investigation in the discussion (p.13).

Regarding the models accommodating other stimuli – while the primary focus of this study utilized edge-orientation as the choice stimulus to test discrimination accuracy, our simulation and models can be readily used to study the encoding other stimuli such different geometric shapes or textures, and different stimulus strengths/indentations. We added a point to this effect in the discussion (p. 9-10).

3. How does orientation decoding performance compare to previously published performance in a comparable exercise with fundamentally different encoding and decoding models (Delhaye et al., JNP, 2018)?

As noted above, our interest was not in comparing model performance per se but rather in trying to gain some mechanistic insight into how the underlying biology. We added a discussion about the comparison to the study the reviewer mentioned. Specifically, while the authors decoded edge-orientation from first-order neurons by inputting their activity into machine learning tools, our study examined the decoding afforded by synaptic integration, to better gauge the computational power afforded by physiological synaptic mechanisms rather than quantify the theoretical decoding limits (p.13-14).

Minor comments

Does it make sense to run an analysis of performance at different time windows with “NMDA” currents given their long integration times?

We performed the analysis using NMDA synapses to complement the analysis conducted using AMPA synapses. As the reviewer commented, the long integration time suggests that the NMDA would allow robust discrimination, however we also studied time windows that were larger than the NMDA decay time (> 100 ms, often 500 ms for a full sweep of an edge over the 10x10 mm skin patch.) We have added a comment to this effect in the discussion (p.10-11).
