# Supplementary material for: Orientation processing by synaptic integration across first-order tactile neurons
Source: PLoS Comput Biol. 2020 Dec 2;16(12):e1008303. doi: 10.1371/journal.pcbi.1008303 (PMC7710081; doi:10.1371/journal.pcbi.1008303)
Supplement: Attachment — Submitted filename: Hay 2020 - response to reviewers.pdf [file pcbi.1008303.s002.pdf]

Reviewer #2: I am happy with the revisions. My initial set of concerns have been adequately addressed in the revised manuscript and in the response to reviewers. My remaining points are all quite minor.

We thank the reviewer for their comments.

-The author summary seems like recycled version of Abstract. It would be helpful to use the Author Summary to describe the main findings and their impact, rather than re-hashing the approach, as already outlined in the Abstract.

According to the reviewer's suggestion, we revised the Author Summary to focus less on the technical approach and instead better describe the main findings and their impact.

-Second-to-last paragraph of Introduction: the authors seem to imply that central neurons are upstream of peripheral neurons. I always consider the postsynaptic neuron to be downstream. If there is any room for confusion here, I would suggest replacing "downstream" with "central" or "second-order".

According to the reviewer's suggestion, we have replaced "upstream" with "higher-order" (p.3).

-Figure 1 legend: I think it is clearer to explain that each mechanoreceptor receives its own "axonal branch", as opposed to its own "axon", as those various branches come together to form a single axon projecting to the CNS. This terminology comes up in a couple other places, and though minor, the suggested change might help avoid confusing certain readers.

According to the reviewer's suggestion, we have rephrased to "axonal branch" in the legend of Figure 1 (p.4), and in the results (p.3), methods (p.15)

-Above Figure 3 legend: "…increased the robustness of discrimination of classifiers [that] used fast-decaying…"

We have revised to add the missing word "that".

-Figure 2 legend: "…each of which innervat[ed] a single mechanoreceptor"

Perhaps the reviewer meant Figure 7 legend? We have revised the Fig 7 legend accordingly.

-First paragraph of Discussion; "used to study the encoding [of] other stimuli"

We have to add the missing word "of".

-Fourth paragraph of Discussion: the authors suggest that "differentiation between AMPA and NMDA input streams could be established for example via compartmentalized synaptic locations on the postsynaptic neuron dendrites". It is not clear to me how that would happen if both types of synaptic inputs evoke spikes that are interspersed in the same neuron. I appreciate how the authors might decode using the PSP amplitude in different dendritic branches, but that is ultimately not a biologically realistic decoding scheme. Could the authors please clarify this idea.

We clarified this discussion point at the level of synaptic input and the level of the spike output (p.10-11):

The differentiation between AMPA and NMDA input streams could be established for example via NMDA spikes triggered by particular input combinations in compartmentalized synaptic locations on the postsynaptic neuron dendrites [17]. In turn, the resulting outputs can be combined into a single spike train by multiplexed neural coding, whereby the two input streams yield spike trains with different frequency phase-locking or levels of synchronicity and can thus be multiplexed [18].

The authors state that the model code will be made available. I think that is what's critical for this study.

Regarding the models, code and data availability, we have added the ModelDB accession number for the entry, which will be made public upon publication.

Reviewer #3: I'm satisfied with the authors revisions.

We thank the reviewer for his comments.

One question: Can we glean, from this study, a prediction about the RF structure of cuneate neurons? Is that what is shown in Figures 4 and 5C?

The results in Figures 4 and 5 indeed provide a prediction about the RF structure of second-order neurons, in the context of discriminating orientations. This prediction could be further refined in the future by examining synaptic integration for discriminating more orientations simultaneously, or discriminating other types of stimuli such as geometric shapes. We have added this point to the relevant discussion paragraph (p.12).
